# Risk factors of visceral leishmaniasis: a case control study in north-western Ethiopia

**DOI:** 10.1186/s13071-014-0470-1

**Published:** 2014-10-14

**Authors:** Solomon Yared, Kebede Deribe, Araya Gebreselassie, Wessenseged Lemma, Essayas Akililu, Oscar D Kirstein, Meshesha Balkew, Alon Warburg, Teshome Gebre-Michael, Asrat Hailu

**Affiliations:** Aklilu Lemma Institute of Pathobiology, Addis Ababa University, Addis Ababa, Ethiopia; Department of Biology, College of Natural Science, Jigjiga University, Jigjiga, Ethiopia; Brighton & Sussex Medical School, Falmer, Brighton, UK; School of Public Health, Addis Ababa University, Addis Ababa, Ethiopia; Department of Zoological Science, Addis Ababa University, Addis Ababa, Ethiopia; Department of Microbiology and Molecular Genetics, The Institute of Medical Research Israel-Canada The Kuvin Center for the Study of Infectious and Tropical Diseases, Faculty of Medicine, The Hebrew University, Hadassah Medical School, Jerusalem, Israel; Department of Microbiology, Immunology and Parasitology, Faculty of Medicine, Addis Ababa University, Addis Ababa, Ethiopia

## Abstract

**Background:**

Visceral leishmaniasis (VL, also called ‘’kala-azar”), is a life threatening neglected tropical infectious disease which mainly affects the poorest of the poor. VL is prevalent in Ethiopia particularly in the northwest of the country. Understanding the risk factors of VL infection helps in its prevention and control. The aim of the present study was to identify the factors associated with VL.

**Methods:**

A case–control study was carried out during the period of January-July 2013 in northwest Ethiopia. Cases and controls were diagnosed using clinical presentation, the rk39 rapid diagnostic test and Direct Agglutination Test (DAT). A total of 283 (84.8% males versus 15.2% females) participants were interviewed. 90 cases and 193 controls were involved, matched by age, sex and geographical location with a ratio of 1:2 (case: controls). Univariate and backward multivariate conditional logistic regression were used to identify risk factors of VL.

**Results:**

Elevated odds of VL was associated with goat ownership (OR = 6.4; 95%: confidence interval [Cl]: 1.5-28.4), living in houses with cracked wall (OR = 6.4; 95% Cl: 1.6-25.6), increased family size (OR = 1.3; 95% Cl: 1.0-1.8) and the number of days spent in the farm field (OR = 1.1; 95% Cl: 1.0-1.2). However, daily individual activities around the home and farm fields, mainly sleeping on a bed (OR = 0.2; 95%: Cl 0.03-0.9), sleeping outside the house under a bed net (OR = 0.1; 95% Cl: 0.02-0.36)] and smoking plant parts in the house during the night time (OR = 0.1; 95% Cl: 0.01-0.6) were associated with decreased odds of being VL case.

**Conclusion:**

Our findings showed that use of bed net and smoke could be helpful for the prevention of VL in the area particularly among individuals who spend most of their time in the farm. VL control effort could be focused on improving housing conditions, such as sealing cracks and crevices inside and outside houses. Further research is warranted to elucidate the role of goats in the transmission of *L. donovani*, assess the impact of bed nets and the role of the traditional practice of smoking plants.

## Background

Visceral leishmaniasis (VL, also called ‘’kala-azar”), is a life threatening neglected tropical infectious disease which mainly affects the poorest of the poor. VL, like HIV, suppresses the immunity of infected individuals and is fatal if left untreated. It is transmitted from human to human by the bite of female phlebotomine sand flies. More than 90% of global VL cases occur in six countries: Bangladesh, Brazil, Ethiopia, India, South Sudan and Sudan [1]. East Africa is the second largest VL focus in the world affecting several communities in Sudan, South Sudan, Kenya, Uganda, Somalia and Ethiopia [1,2].

VL is prevalent in Ethiopia particularly in the lowlands of the country where it is suitable for breeding habitats of the vectors [3,4]. Annually 3,700-7400 estimated cases occur [5]. It is estimated that 3.2 million people are at risk of VL in Ethiopia [6]. It is endemic in northwest, northeast, southwest and southern parts of the country [4-11]. In recent years, VL has also spread to areas where it was previously non-endemic (Libo-Kemekem and Sheraro) [6,9,11]. Seasonal migration of agricultural labourers between endemic and non-endemic regions, combined with biological, environmental and socio-economic risk factors may be responsible for the spread of this disease. Factors such as dog ownership, sleeping under an *Acacia* tree during the day, sleeping outside at night time and poor housing conditions were identified as increased VL risk in an epidemic area of Libo-Kemkem district, Amhara region [11].

Several VL cases have been reported from Kafta Humera district, northwest Ethiopia since the 1970s [3,6,12]. Anema and Ritmeijer [3] reported that more than 6000 cases were treated in Kafta Humera district from 1997 to 2004. VL outbreak has occurred throughout the Humera lowland since 1995, Mykadra village being one of the hot spots [4]. People who live in settlement areas and migrant workers are the most vulnerable to VL in the endemic region. For instance, from 1997 to 2004 more than 80% of patients with VL were male migrant workers infected with *L. donovani* who sleep in the farm [3,4]. Kafta Humera and its surrounding areas have significant economic input for the country because cash crops such as sesame, cotton and sorghum are grown at a commercial scale. Due to this, hundreds of thousands of male migrant workers arrive every year in this place during the agricultural season (June-November) [3,13]. The migrant workers are at high risk of acquiring VL as they live and work being exposed to vectors of VL.

*Phlebotomus orientalis*is a major vector of *L. donovani* in eastern Sudan [14] and it is the most likely vector in northwest Ethiopia [15,16]. This vector is mainly associated with cracked vertisols (black cotton soil) and *Acacia-Balanite* forests [17,18]. *P. orientalis* is the most predominant species and active during the night time and is more abundant during the dry season in the agricultural field and vicinity of the resettlement village [16]. However, a disease control strategy is still unpractical because the reservoir host and the ecology and behaviour of the vector have not yet been fully explicated. Therefore, identifying the risk factors of visceral leishmaniasis transmission and understanding the knowledge, attitude and practices that affect transmission of VL is vital to designing appropriate control methods to reduce the burden of cases in the area. The purpose of the present investigation was to assess the knowledge of villagers about kala-azar and to identify the risk factors associated with VL cases using a case–control study.

## Methods

### Study area and population

This study was carried out from the period of January to July 2013 in the western Tigray region, northwest Ethiopia, which borders Eritrea to the north and Sudan to the west. Based on the 2007 national census, Kafta Humera district has a total population of 92,167. Of these, 32.8% are urban inhabitants while the rest live in semi-urban and rural areas. Kafta Humera has been known as the focus of VL since 1970 [12]. We selected the study area based on recent VL cases diagnosed in Kahsay Abera Hospital, Setit Humera. The study participants are settlers in Adebay village who returned from Sudan in 1993. The communities in Endris/Hagereselam are also settlers who originated from different parts of Tigray region in the 2003 resettlement program. These two localities comprise more than 10,000 individuals. Agriculture comprises the major economic activity of the fertile black cotton soil (vertisol), supporting cultivation of sesame, cotton and sorghum. The region attracts a large number of labourers especially during the weeding and harvesting period with an average of 200,000 workers migrating each year from different parts of Ethiopia and Sudan [19]. Recently VL cases were highly prevalent in resettled communities and male migrant workers. It seems the area is more conducive for the availability of the vector and reservoir host. Cracked vertisol and forests (dominated by *Balanites* spp.) are more abundant as compared to other areas in the district [18].

In the study area, only Kahsay Abera hospital provides diagnostic and treatment services pertained to VL. Adebay and Hagereselam villages are about 20 and 30kms from the urban city of Setit Humera respectively. The villages are located at altitude ranges of 500 m-650meters a.s.l. and along coordinates 14° 11.47’N and longitudes of 36°46.07’E. Temperature reaches an average of 42°C between April and June and falls to between 25 and 35°C during the months between June and February. The average annual rainfall is 400-650 mm; with July and August receiving the highest amounts and rain is absent between October and April [20].

### Research design

A retrospective case–control study was carried out based on visceral leishmaniasis cases, a total of 1128 VL cases were admitted and treated at Kahsay Abera Hospital from July 2011 to August 2013. These patients came from Setit Humera, Kafta Humera, Welkayit and Tsegede districts of northwest Ethiopia. Only 90 cases were included in the case and control study. VL patients came from Adebay and Endris/Hagereselam locality (administered under Kafta Humera district). Information about patients; i.e., age, sex and place of residence, date of admission, clinical features on admission and outcomes were collected from the registry of the hospital. Information relating to the patients, such as name, age, address, (Tabia/Kebele) is routinely recorded in the Registration Department. Patients were diagnosed by physicians at this Hospital and defined as a person having fever for at least 2 weeks, associated with weight loss and/or splenomegaly and confirmed either parasitologically or by rk39 dipstick test. Most of the case patients were treated before this study began (2011–2013).

For each case we recruited two controls without VL case history from the left or right side of the neighbouring house of the VL case. We only recruited cases the most recent one when VL treated more than one occurred in a household.

Each control was matched to the respective case by sex, location, migrant worker and age range (<5 years, 5–14 years, 15–39 years, and 40 years of age or older). In case no one matched the criteria, we used the next house. We recruited migrants for case control study individuals living in the area temporarily for agricultural practices. The respondents were interviewed by trained health professionals. Cases and controls responded to the interviews using a structured questionnaire to capture data regarding their demographic and socio economic characteristics (age, gender, occupation, education, time of living in village and family size), house construction material and its condition, domestic animal ownership and their number per house, kinds of animals kept, sleeping habits, use of bed net and individual activity in the agricultural fields. The questionnaire was also comprised of questions relating to the respondents’ knowledge, attitudes and common practices towards VL and sand flies. The questionnaires was translated into the local language (Tigrigna) and pre-tested.

### Sample size determination

The sample size was calculated according to dog ownership and we chose the one with higher sample size in the study. Since owning a dog was found to be a risk factor for VL in a previous study in Amhara region [11] where it was found that 37.4% of controls had dogs. To detect an odds ratio of 2.28, with 80% power and 95% CI and 1:2 cases to control ratio, the final sample size was estimated at 78 cases and 155 controls. Adding 10% non-response rate the final sample size was 86 cases and 172 controls, with a minimum sample size of 258.

### Direct Agglutination Test (DAT)

Asymptomatic VL infections usually occur in high endemic areas. However, blood samples were collected from the control groups, which were selected primarily on the basis of the absence of clinical symptoms and had no history of VL. Blood collection was carried out by health professionals recruited to the project. The serum was separated at Setit Humera Health Centre Laboratory and transported in a cool box to Addis Ababa. The serum was diagnosed by DAT at Addis Ababa University, Medical Faculty, Leishmaniasis Research & Diagnostic Laboratory. Serologic tests were carried out to identify asymptomatic infections among the controls.

DAT is a simple and user friendly test. The estimated sensitivity of DAT was 94% in Ethiopia [21]. In performing DAT, serum samples were diluted in physiological saline (0.9% NaCl) containing 0.8% β-mercaptoethanol. Two-fold serial dilutions of the sera were made, starting at a dilution of 1:100 and going up to a maximum serum dilution of 1:102,400. Freeze-dried DAT antigen produced by KIT Biomedical Research was reconstituted with physiological saline. 50 μL of DAT antigen solution (concentration of 5 × 10^7^parasites per ml) was added to each well containing 50 μl of diluted serum. The results were read after 18 hours of incubation at ambient temperature. The cut-off value was established considering the titres obtained in samples from negative controls. Therefore, a sample was considered positive if it had a titre of 1:800 and above.

### Ethical statement

Ethical clearance was obtained from Addis Ababa University, Medical Faculty, Department of Microbiology, Immunology and Parasitology. The study was part and parcel of a larger study on VL transmission dynamics supported by the Bill & Melinda Gates Foundation: “Studies on the ecology and transmission dynamics of visceral leishmaniasis in Ethiopia”. This project obtained ethical approval from the National Ethics Review Board. Support letters were obtained from the western Tigray Zone and Kafta Humera district Health Bureaus. Informed consent was obtained from all cases and controls, for young children under 18 years old, consent was obtained from their parents.

### Data analysis

Data were entered and analyzed by the statistical packages SPSS, release 16 (SPSS Inc., Chicago, IL, USA) and STATA version 13(Stata Corporation, College Station, TX, U.S.A.). For descriptive statistics, frequency, percentage and mean were used. Association between dependent and independent variables were measured by use of Chi-square test. The risk factor data was analyzed by univariate and backward multivariate conditional logistic regression using the age, sex and residence matching. Odd ratio was calculated with 95% confidence interval for each variable. Variables with p < 0.10 in the univariate analyses were consequently tested in multivariable models. All statistical tests were carried out at a significance level of 0.05.

## Results

### Retrospective data

**Figure 1:**
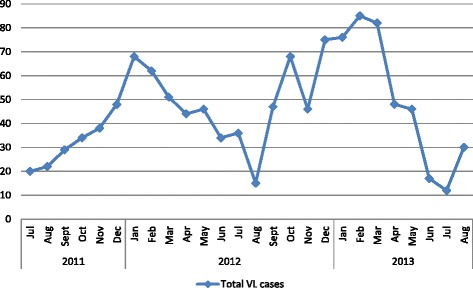
**Monthly VL admitted and treated at KahsayAbera Hospital, NW Ethiopia.**

**Figure 2:**
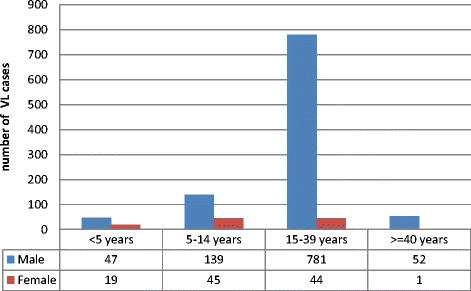
**Distribution of VL cases by age and sex, amongst VL patients from KahsayAbera Hospital, Northwest Ethiopia, July 2011– August 2013.**

A total of 1128 (1019 males: 109 females) VL patients were admitted and treated at Kahsay Abera Hospital from the period of July 2011 to August 2013. The diagnoses were based on the patients’ history, clinical examinations, parasitological investigations, and/or rk39 dipstick test results. The number of VL cases varied over the seasons, with a peak in January and February (dry season) sharply declined during the wet season (June, July, and August) (Figure 1). Figure 2 shows distribution of VL cases by age and sex. Most of the cases(825) were in the age group 15–39 years; the second affected age groups were 5–14 years (184 cases) followed by <5 years (66 cases) and > = 40 years (53 cases).

### Case–control study

#### Socio demographic characteristics

A total of 283 (84.8% males and 15.2% females) were involved in the study, of which 90 were cases (85.6% males versus 14.4% females) and 193 were controls (84.5% males and15.5% females), matched by age, sex and location (case: control ratio of 1:2). For some of the cases, there were more than two controls. Blood samples were taken from 193 control participants, and tested using DAT; and all samples were found to be negative for *L. donovani* infection. Cases and controls were comparable with respect to many characteristics, except cases tended to have larger family size compared to controls (p = 0.023); controls tended to be from Tigray ethnic groups compared to cases (p = 0.026). In addition, the two groups significantly differed with respect to occupation, educational status and duration slept in the field as described in Table 1.

**Table 1:** **Socio demographic characteristics of participants in case–control study in the visceral leshmaniasis endemic foci of the Adebay and Hagereselam, North -West Ethiopia**

**Characteristics**	**Cases no. (%)**	**Control no. (%)**	**Total**	**P value**
Gender				0.810
Male	77(85.6%)	163(84.5%)	240(84.8%)	
Female	13(14.4%)	30(15.5%)	43(15.2%)	
Age in years				0.975
<5	3(3.3%)	6(3.1%)	9(3.2%)	
5-14	24(26.7%)	49(25.4%)	73(25.8%)	
15-39	56(62.2%)	120(62.2%)	176(62.2%)	
> = 40	7(7.8%)	18(9.3%)	25(8.8%)	
Relation to household head				0.973
Head	22(24.4%)	55(28.5%)	77(27.2%)	
Spouse	5(5.6%)	13(6.7%)	18(6.4%	
Female child	7(7.8%)	15(7.8%)	22(7.8%)	
Male child	45(50.0%)	88(45.6%)	133(47.0%)	
Distantly related	6(6.7%)	13(6.7%)	19(6.7%)	
Others (dependent, servant)	5(5.6%)	9(4.7%)	14(4.9%)	
Current marital status				0.334
Single	61(67.8%)	129(67.2%)	190(67.4%)	
Married	28(31.1%)	63(32.8%)	91(32.3%)	
Divorced	1(1.1%)	0(.0%)	1(.4%)	
Family size				0.025
1-2	9(10.0%)	36(18.7%)	45(15.9%)	
3-5	29(32.2%)	76(39.4%)	105(37.1%)	
6-8	39(43.3%)	69(35.8%)	108(38.2%)	
> = 9	13(14.4%)	12(6.2%)	25(8.8%)	
Ethnic group				0.026
Tigre	83(92.2%)	188(97.4%)	271(95.8%)	
Amhara	4(4.4%)	5(2.6%)	9(3.2%)	
Others	3(3.3%)	0(.0%)	3(1.1%)	
Major occupation currently do earn money				0.045
Employed	0(0%)	1(.5%)	1(.4%)	
Business man/women	3(3.4%)	6(3.1%)	9(3.2%)	
Farmer	25(28.1%)	56(29.3%)	81(28.9%)	
Housewife	8(9.0%)	13(6.8%)	21(7.5%)	
Daily labourer	15(16.9%)	29(15.2%)	44(15.7%)	
Student	27(30.3%)	73(38.2%)	100(35.7%)	
Have no Job	1(1.1%)	9(4.7%)	10(3.6%)	
Others (keeping Animal)	10(11.2%)	4(2.1%)	14(5.0%)	
Able to read and write in any language (Yes)	60(66.7%)	144(74.6%)	204(72.1%)	0.165
School grade completed				0.001
Grade <5	60(66.7%)	89(46.1%)	149(52.7%)	
Grade > =5	30(33.3%)	104(53.9%)	134(47.3%)	
Permanent residence				0.676
Rural	17(18.9%)	30(15.5%)	47(16.6%)	
Semi-urban	72(80.0%)	157(81.3%)	229(80.9%)	
Urban	1(1.1%)	5(2.6%)	6(2.1%)	
Study area (villages)				0.888
Adebay	69(76.7%)	152(78.8%)	222(78.1%)	
Hagereselam/Endris	10(11.1%)	18(9.3%)	28(9.9%)	
Migrant workers	11(12.2%)	23(11.9%)	34(12.0%)	
Own farm land (Yes)	66(73.3%0	140(72.5%)	206(72.8%)	0.889
Months sleep in the farm field				0.000
June-November	55(77.5%)	104(95.4%)	159(88.3%)	
December-May	0(.0%)	5(4.6%)	5(2.8%)	
Throughout the year	16(22.5%)	0(.0%)	16(8.9%)	

#### Knowledge, attitude and practices

All the 283 of participants were interviewed about their awareness of VL. If a child (<15 years old) was a case or a control, parents were interviewed on behalf of their children. Of the total participants, most (94.0%) had previously heard about the disease (Table 2).

**Table 2:** **Knowledge of the participants on VL and sand flies vector**

**Variables**	**Frequency**	**Percent (%)**
Knowledge		
knowledge on VL		
Awareness about kala-azar	266	94.0%
Know the signs and symptoms of kala-azar	171	60.4%
Mode of transmission		
By the bite of sand fly	85	30.0%
By the bite of mosquitoes	148	52.3%
Do not know	50	17.7%
Knowledge on sand fly		
Identify or recognize the vector	30	10.6%
Know about the breeding habitat of the vector		
Dirty place	41	14.5%
Cervices in the house	20	7.1%
Thatched roof	2	.7%
Damp and dark areas	51	18.0%
*Acacia-Balanite* trees	70	24.7%
Cracked black cotton soil	21	7.4%
Sand soil	22	7.8%
Near animal burrow	14	4.9%
Do not know	42	14.8%

Concerning attitudes about severity of VL, 64.3% of the respondents considered VL as a more severe disease compared to malaria or other diseases (Table 3). The majority (94.7%) of the participants preferred modern medicine for treatment of VL. The study participants were practicing different methods to prevent mosquitoes and other biting flies; 69.9% of the respondents used a bed net; 15.9% cleaned their environment; while 8.5% used insecticide spraying and 5.3% used repellents (Table 3). Friends and neighbours (46.6%) and health personnel (45.6%) were major sources of information about kala-azar in the study area. The mass media such as television (4.2%), radio (1.4%) and newspapers (0.7%) were indicated as limited information sources. Similarly the school (1.4%) was also identified as a limited source of information (Table 3).

**Table 3:** **Attitude and practice of the participants on VL and sand flies vector**

**Variables**	**Frequency**	**Percent (%)**
**Attitude**		
The degree of severity of kala-azar as compared to malaria, kala-azar is		
Very serious	182	64.3%
Serious	95	33.6%
Ordinary	6	2.1%
Kala-azar is an important problem of disease		
Yes	227	80.2%
No	56	19.8%
Kala-azar can be controlled through community participation		
Yes	131	46.3%
No	152	53.7%
Kala-azar can affect family income		
Yes	252	89.0%
No	31	11.0%
Kala-azar is fatal disease if it is untreated		
Yes	280	98.9%
No	3	1.1%
**Practice**		
Drug preference for treatment VL		
Specific medicine	268	94.7
Indigenous medicine	4	1.4
Do not know	11	3.9
Prevention measures from mosquito/sand fly bite		
Use of bed net	197	69.6
Insecticide spraying	24	8.5
Repellents	15	5.3
Cleanliness	45	15.9
Do not know	2	.7
Source of information		
Health personnel	129	45.6
Friends and neighbours	132	46.6
Television	12	4.2
Magazines	2	.7
Radio	4	1.4
School	4	1.4

### Risk factors

Table 4 depicts factors associated with VL transmission in univariate analysis. Factors associated with boosted VL odds were: family size in household, education level below grade five, damp house floors, cracked house walls and cracked black soil near houses. Similarly, animal ownership such as presence of cattle, owning dogs, goats and owning donkeys were found to significantly increase the odds of VL. Individual behaviour in the domestic and in the farm fields like dumping animal dung near houses, sleeping outside the house near animal shelters, sleeping under *Balanites* and *Acacia* trees at night, increased the number of days spent in the farm field and sleeping in the farm field over night was also associated with higher odds of VL risk.

**Table 4:** **Factors associated with transmission of visceral leishmaniasis in univariate analysis**

**Variables**	**Cases**	**Control**	**OR**	**95.0% CI**		**P value**
	**[n/N (%)]**	**[n/N (%)]**		**Lower**	**Upper**	
**Socio-demographic**						
Family size (mean, 95% Cl)**	5.8(5.3-6.4)	4.8(4.4-5.1)	1.257	1.110	1.423	0.000
Own farm land	66(73.3%)	140(72.5%)	1.038	0.555	1.943	0.907
Able to read and write	60(66.7%)	144(74.6%)	0.598	0.319	1.121	0.109
School grade <5	60(66.7%)	89(46.1%)	3.432	1.760	6.693	0.000
**Housing condition**						
Separate kitchen	43(47.8%)	122(63.2%)	0.534	0.320	0.892	0.016
Animal barn inside compound	45(50.0%)	79(40.9%)	0.622	0.350	1.106	0.106
Damp house floor	24(26.7%)	15(7.8%)	4.328	2.097	8.932	0.000
Sleeping on bed	74(82.2%)	183(94.8%)	0.287	0.128	0.642	0.002
Thatched roof	56(62.2%)	113(59.5%)	0.899	0.523	1.546	0.701
Cracked house wall	60 (66.7%)	84 (44.4%)	2.768	1.549	4.946	0.001
Cracked black soil near house	64(71.1%)	60(31.1%)	6.266	3.308	11.871	0.000
**Animal ownership**						
Presence of cattle (mean, 95% Cl for mean)**	5.9(3.4-8.3)	1.8(1.3-2.3)	1.114	1.047	1.185	0.001
Dog ownership	47(52.2%)	48(24.9%)	3.544	1.970	6.376	0.000
Goats ownership	37(41.1%)	43(22.3%)	2.500	1.407	4.443	0.002
Number of goats per household (Mean,95% Cl for mean)**	4.01(2.4-5.6)	2.1(1.3-3)	1.041	1.002	1.083	0.039
Sheep ownership	38(42.2%)	72(37.3%)	1.183	0.710	1.971	0.519
Number of sheep per household (Mean,95% Cl for mean)**	3.9(2.1-5.6)	3.4(2.4-4.5)	1.006	0.975	1.038	0.704
Donkey ownership	59(65.6%)	98(50.8%)	1.971	1.117	3.477	0.019
Dumping animal dung near house	38(42.2%)	30(15.5%)	4.170	2.215	7.849	0.000
Apply insecticide to livestock	21(23.3%)	64(33.2%)	0.603	0.339	1.072	0.085
**Individual activities**						
Sleeping outside the house near animal shelter	43(47.8%)	46(23.8%)	3.062	1.725	5.437	0.000
Sleeping under *Balanites*-*acacia* trees at night	64(71.1%)	96(49.7%)	2.613	1.453	4.702	0.001
Sleeping outside the house under bed net	16(17.8%)	118(61.1%)	0.130	0.066	0.258	0.000
House sprayed with insecticides	16(17.8%)	49(25.5%)	0.577	0.290	1.148	0.117
Smoking house	50(56.2%)	168(87.0%)	0.093	0.038	0.225	0.000
Period of days stayed in the farm field						
Mean ( 95% Cl for mean)**	91.3(61.7-120.9)	8(5.9-10)	1.035	1.009	1.062	0.008
Sleeping in the farm field over night	66(76.7%)	109(56.5%)	4.159	1.745	9.913	0.001

Odd ratios (OR), 95% confidence intervals (Cl), and P values derived from univariate conditional logistic regression models. **refers to calculated means.

On the other hand, having a separate kitchen, sleeping on a bed, sleeping outside the house under a bed net during the warm season and smoking plant parts inside and outside houses had decreased odds of VL.

Table 5 depicts factors associated with VL in multivariable conditional logistic regression models. The odds for VL consistently increased as the size of the family increased in the household (OR1.3; 95% Cl: 1.026-1.799). Individuals who lived in a house with cracked walls had 6 times increased odds of being infected with VL compared with their counterparts (OR 6.495% Cl; 1.585-25.580). Similarly, those who owned a goat had increased odds of a being VL case compared with those who did not own goats (OR 6.4; 95%: Cl 1.5-28.4). However, daily individual activities around the home, mainly sleeping on a bed (OR 0.2; 95%: Cl 0.03-0.9)], sleeping outside the house under a bed net (OR 0.093 (95% Cl 0.024-0.357)] and smoking plant parts in the house during the night time [OR 0.082 (95% Cl 0.011-0.630)] were associated with decreased odds of being a VL case. Finally, the risk for VL was always elevated as the number of days spent in the farm field increased [OR 1.100 (95% Cl 1.015-1.192)].

**Table 5:** **Factors associated with VL in a ‘multivariate conditional logistic regression model among the community in Northwest Ethiopia**

**Variables**	**Cases**	**Controls**	**Adjusted OR ratio**	**P value**
	**No. (%)**	**No. (%)**	**(95% Cl)**	
Family size (mean 95% Cl)	5.8(5.3-6.4)	4.8(4.4-5.1)	1.359(1.026-1.799)	0.032
Sleeping on bed	74(82.2%)	183(94.8%)	0.158(0.028-0.898)	0.037
Ref. ground	16(17.8%)	10(5.2%)	1	
Cracked house wall (Yes)	60 (66.7%)	84 (44.4%)	6.368(1.585-25.580)	0.009
Ref. (un-cracked)	30(33.3%)	105(55.6%)	1	
Own goats (Yes)	37(41.1%)	43(22.3%)	6.445(1.463-28.384)	0.014
Ref. (No)	53(58.9%)	150(77.7%)	1	
Sleeping outside the house under bed net	16(17.8%)	118(61.1%)	0.093(0.024-0.357)	0.001
Ref. (without bed net)	74(82.2%)	75(38.9%)	1	
Smoking house (Yes)	50(56.2%)	168(87.0%)	0.082(0.011-0.630)	0.016
Ref. (No)	39(43.8%)	25(13.0%)	1	
Number of days stayed in the farm field (mean 95% CL)	91.3(61.7-120.9)	8(5.9-10)	1.100(1.015-1.192)	0.020

Odd ratios (OR), 95% confidence intervals (Cl), and P values derived from multivariable conditional logistic regression models.

## Discussion

Identifying the risk factors of VL infection in northwest Ethiopia offers a significant basis of information to design and develop effective control measures. There is poor knowledge, attitude and practice gap towards VL in the study area. Our results showed the presence of goats, larger family size; individual houses with cracked walls and number of days spent in the farm field were associated with increased risk of VL infection. On the other hand, daily individual behaviour in the domestic and natural habitat; mainly sleeping on a bed, sleeping outside the house with a bed net and smoking plant parts in the house at night time were associated with reduced risk of VL infection. The results here were in conformity with previous studies [22-34].

A total of 1128 (1019 males: 109 females) VL patients were admitted at Kahsay Abera Hospital during the period of July 2011 and August 2013. These patients came from different districts of northwest Ethiopia; mainly from Setit Humera, Kafta Humera, Welkayit and Tsegede. From the data, more male patients were seen than female patients, with a male to female ratio of 9:1. A similar result was reported in Gondar and Axum hospitals [35,36]. This high male patient load could be due to the economic activities that entail gender bias. Previous studies documented that males are disproportionately affected by VL compared to females, this is mainly related to their work (agricultural activities, keeping animals, daily labourer and soldiers) [35,36].

The present study revealed that VL case load in the hospital peaked during January and February, the dry season in the area. This was consistent with the agricultural labourer movement that peaks during the period June-November. In south Ethiopia, a high number of VL patients were diagnosed during August to November (rainy season) [37]. The VL transmission seasons (months) in Kafta Humera lowlands have not yet been delineated. However, its transmission may be associated with the abundance of sand flies in the area (March, April, May and early June) [Yared *et al*., unpublished]. Higher number of VL cases were recorded above 14 years of age group, which is in contrast to another study in South Sudan, where 56% of the cases were under 5 years old [38].

Almost all the study participants identified or have heard of VL. This is in agreement with studies conducted elsewhere in endemic communities [39-41]. The current study area has been known to be endemic for VL for more than four decades [12]. However, only a few of participants knew the mode of transmission and recognized the vector. Besides, only 7.4% of the participants knew the correct breeding sites of sand fly vectors in the area. However, recent study showed that cracked vertisols were identified as breeding site of *P. orientalis* and other sand flies in northwest Ethiopia [18]. As a result our observation indicated that enhancing awareness about specific breeding habitats of sand flies and mode of VL transmission are very vital to reduce the transmission of the infection.

In this study, owning goats was associated with elevated VL risk. Perhaps goats play no role as reservoirs of *L. donovani*, but they may attract the vector to human houses and thereby increase the risk of human exposure to infected sand flies. In the present study area, entomological studies showed that *P. orientalis* (the presumed vector of VL in north Ethiopia) has a feeding preference to domestic animals [16]. Similarly, owning domestic animals (cows, buffaloes or goats) was associated with a higher risk of being DAT positive VL elsewhere [23,30,32,33,42-44]. Another study in Ethiopia (Libo Kemekem district) has revealed that dog ownership in the villages was a significant risk factor for VL [11]. Studies carried out in Nepal and Bangladesh, found out that ownership of large domestic animals such as cattle and water buffalo was strongly protective [22,24]. On the other hand, a case–control study in Bihar (India) found no significant associations between VL and keeping domestic animals inside the house or ownership of domestic animals [30]. Thus, different studies in different endemic countries have reported different or similar domestic animals as either risk factors or protective. This could be due to the variation of geographical location, the abundance of the host, the abundance, feeding preference, breeding habitat and resting site of the vector and the leishmanial infection rate.

We found a very strong association between VL and poor housing conditions, such as cracked house walls. Cracked walls could be made through drying and could be used as ideal breeding and resting sites of sand flies. We collected *P. orientalis* from the exterior cracked wall of houses using sticky traps in the villages (Yared *et al*. unpublished). Our study confirmed individuals who are sleeping regularly near the cracked walls are at higher risk of VL. In southern Ethiopia, an un-plastered house was a major individual risk factor [45]. Similarly in Nepal, houses constructed in mud and thatched with a damp floor were associated with elevated risk of VL [22,26,34]. In India, mud-plastered walls in houses and houses not sprayed with DDT in the past six months were significant risk factors for kala-azar [25,30]. In India and Kenya [27,29] mud plastered wall, mixed dwelling and cattle shed were found to be ideal environments for phlebotomine sand flies.

The final logistic regression model in the present analysis revealed larger family size in the household was associated with elevated VL risk. Two studies in Nepal agree with our result [26,34]. This could be due to the increasing amount of carbon dioxide and odours released in the homestead that attract a large number of sand flies. Similar to previous studies [22,34], our results showed sleeping on a bed was more protective as compared to sleeping on the ground. Studies indicated that sand flies emerge from the cracked vertisol [18] and move in short hops and fly close to the ground [46]. It also indicated that the risk for VL increased as the number of days spent in the farm fields increased. *P. orientalis* was found to be abundant in the farm fields (Yared *et al.,* unpublished) and this may indicate VL transmission occurs outside of the villages. In Nepal, a regular forest visit was also associated with elevated VL odds [34]. Put together, these findings are highly indicative of the need for policy makers to devise control methods not only in the villages, but in the farm fields as well.

Bed nets were associated with protection of VL in Bangladesh and Nepal [22,24]. A case control study in East Africa has also revealed that owning a mosquito net was associated with a reduced risk of VL [28]. Similarly our result confirmed that sleeping outside houses under bed nets was associated with decreased VL risk. Use of a bed net is significantly important to bar the transmission cycle of *L. donovani* infection from sand fly to human. During the dry season, almost all individuals in the communities sleep outside the house due to the hot weather conditions. Sand flies were also abundant during this time when collected from the vicinity of the villages in the cracked black soil and periphery of villages (Yared *et al*., unpublished). Long lasting insecticide treated nets (LLINs) were distributed by the government and are also commercially available. Bed nets that were not impregnated with insecticide or in poor condition were not protective [26]. Thus bed nets should be impregnated with insecticide in order to enhance its protectiveness.

Smoking plant parts in the house during the night time was associated with strong protection. The villagers, especially during the rainy season, commonly practiced smoking to repel mosquitoes and other night active flies, using wood of Weyiba (a Tigrigna term for *Terminalia brownii* -Combretaceae) inside and outside the house. However, its repellent activities against biting insects, have not been evaluated, and need further investigation. This species is a medicinal plant with antiplasmodial and antimicrobial activities [47]. Due to the exophagic and crepuscular behaviour of *P. orientalis*, LLINs may provide incomplete protection from bites [16]. Complementary control methods, such as repellents, are very vital where vectors are exophagic. Natural repellents such as neem and chinaberry seed oils are grown widely in the region and could be ideal and cheaper repellents [48]. They can be applied to people who work outdoors at night when the sand fly biting peaks, either during early evenings before people retire to bed or when people sleep outside their houses during the warmer seasons and while harvesting at night time.

The major strength of this study was the matching of cases with two controls; taking sex, age group, migration and location as matching variables. Misclassification bias is low since both cases and controls were selected after proper diagnostic tests were performed. We collected blood samples from all controls and tested using DAT to identify asymptomatic *Leishmanial* infection in the control group. However, this study is not without limitation. First income was not measured, because they didn’t know their own monthly and annual income. In addition, we didn’t consider the nutritional status of the respondents. Despite the limitations mentioned here we found very strong indicators of risk factors for VL and these variables would be pivotal for designing appropriate control measures.

These findings have important practical implications because several relevant features that will help to understand VL transmission dynamics have been elucidated. Besides, this study will be helpful for designing and developing effective control methods. It is also supportive for the policy maker to disseminate knowledge at individual and community level and to develop a systematic way of implementing control methods. Future studies considering risk factors of VL should include nutritional status and presence of other animals such as rodents. Additionally, future studies should investigate the risk factors of VL vectors biting at individual level as well as the association with density of phlebotomine sand flies and with the incidence of *L. donovani* infection in human. Finally, future studies should investigate whether these findings are consistent or not.

## Conclusion

In conclusion, we found owning goats, living in houses with cracked walls, large family size and number of days spent in the farm were associated with increased VL risk. In contrast, sleeping on a bed, sleeping outside houses under bed nets and use of smoke at night time was associated with decreased odds of VL. Our findings have implications for planning and control of VL. Firstly, individual protective measures such as use of bed net and smoke could easily be adopted for the prevention of VL in the area particularly among individuals who spend most of their time in the field; and secondly improving housing conditions such as removing cracks from houses would help to reduce risk of VL. There are also research questions to be answered; first, the association of goat ownership and risk of VL should be investigated in detail, second, the repellent effect of the traditional smokes used in the area should be evaluated, and third the effectiveness of available bed nets for prevention of VL should be evaluated.
